# Design, Synthesis, and Electrical Performance of Three-Dimensional Hydrogen-Bonded Imidazole-Octamolybdenum-Oxo Cluster Supramolecular Materials

**DOI:** 10.3390/molecules30153107

**Published:** 2025-07-24

**Authors:** Hongzhi Hu, Adila Abuduheni, Yujin Zhao, Yuhao Lin, Yang Liu, Zunqi Liu

**Affiliations:** 1 Chemistry and Chemical Engineering College, Xinjiang Agricultural University, Urumqi 830052, China; huhongzhi305@163.com (H.H.); 17799751675@163.com (A.A.); 18599215733@163.com (Y.Z.); lin18030922395@163.com (Y.L.); 2 Xinjiang Sub-Center National Engineering Research Center of Novel Equipment for Polymer Processing, Urumqi 830052, China; 3 Xinjiang Key Laboratory of Agricultural Chemistry and Biomaterials, Urumqi 830052, China

**Keywords:** polyoxometalate, supramolecular materials, three-dimensional structures, dielectric anomalies, electrocatalysis

## Abstract

Polyoxometalate (POM)-type supramolecular materials have unique structures and hold immense potential for development in the fields of biomedicine, information storage, and electrocatalysis. In this study, (NH_4_)_3_ [AlMo_6_O_24_H_6_]·7H_2_O was employed as a polyacid anion template, pentacyclic imidazole molecules served as organic ligands, and the moderate-temperature hydrothermal and natural evaporation methods were used in combination for the design and synthesis of two octamolybdenum-oxo cluster (homopolyacids containing molybdenum-oxygen structures as the main small-molecular structures)-based organic–inorganic hybrid compounds, [(C_3_N_2_H_5_)(C_3_N_2_H_4_)][(*β*-Mo_8_O_26_H_2_)]_0.5_ (**1**) and {Zn(C_3_N_2_H_4_)_4_}{[(*γ*-Mo_8_O_26_)(C_3_N_2_H_4_)_2_]_0.5_}·2H_2_O (**2**). Structural and property characterization revealed that both compounds crystallized in the *P*-1 space group with relatively stable three-dimensional structures under the action of hydrogen bonding. Upon temperature stimulation, the [Zn(C_3_N_2_H_4_)_4_]^2+^ cation and water molecules in **2** exhibited obvious oscillations, leading to significant dielectric anomalies at approximately 250 and 260 K when dielectric testing was conducted under heating conditions.

## 1. Introduction

Polyoxometalates (POMs), which are capable of self-organization and possess multilevel structures and excellent electron-accepting properties, are an important component of a series of inorganic functional materials. They have demonstrated broad application prospects in various fields such as photoelectrocatalysis [1,2,3], sensing [4,5,6,7,8], energy storage [9,10,11,12,13,14,15], and medicinal chemistry [16,17]. POMs have a large number of structural coordination sites that can be used for constructing POM-type organic–inorganic hybrid complexes through the modulation of synthesis conditions and design of building blocks. The introduction of organic ligands can enhance their biomolecular compatibility, which make them potentially applicable to targeted drug delivery systems [16,17] and biomedical engineering [18,19,20,21]. In particular, they have demonstrated unique molecular recognition and modulation capabilities in the regulation of antitumor activity and simulation of biomineralization processes [22,23,24,25,26,27]. Homopolyacid compounds have become an important branch of materials in the study of functional POM materials due to the different coordination modes of their transition metals. Since the determination of the [Mo_7_O_24_]^5−^ structure by Sturdivant using single-crystal X-ray diffraction (XRD), researchers have subsequently developed a series of classical Lindqvist-type, Keggin-like, decavanadate, and decatungstate homopolyanions and their derivatives [28,29,30,31]. [Mo_8_O_26_], an important component of homopolymolybdates, most commonly exists in the form of α- and β- isomers. The γ-isomer, which serves as an intermediate in the interconversion of the α- and β-isomers, is less frequently reported; other isomeric forms include the δ-, ε-, ζ-, η-, and θ-isomers [32,33]. In recent years, considerable research effort has been focused on the use of such compounds in the synthesis of octamolybdate–transition metal hybrid compounds. The introduction of transition metal cations into homopolyacids leads to a variety of conformational models in polyacid anions through variable coordination modes. Kortz et al. [34] reported the preparation of a novel homopolyacid compound [H_2_Pt^IV^V_9_O_28_]^5−^ in an aqueous solution by one-pot synthesis using H_2_[Pt(OH)_6_] and NaVO_3_ as raw materials. Structural analysis revealed the presence of an ideal *C*_2v_ symmetric decavanadate structure in which one V^5+^ was substituted by Pt^4+^, while the rest of the octahedral structure was composed of nine {VO_6_}s. This was the first report of the construction of a transition metal-substituted decavanadate derivative.

The spatial flexibility of organic compounds and rigidity of inorganic materials can be incorporated into a single entity via chemical bonding or weak intermolecular forces. This enables the design and synthesis of organic–inorganic hybrid crystalline materials with good bioactivity and optical, electrical, and magnetic properties through approaches such as molecular self-organization and structural coupling [35,36,37,38,39,40]. Such materials also offer various physical property advantages, including flexible and adjustable structures, ease of processing, high strength, and high thermal stability [41,42,43,44,45]. Therefore, they possess broad application prospects in the fields of diversified structural design, catalysis, energy storage, and information storage [46,47,48,49,50]. For instance, a study reported the use of 4-methylpiperidine and CdCl_3_ as raw materials and the implementation of cationic customization and halogen engineering for the preparation of [(4-methylpiperidium)CdCl_3_] with obvious antiferroelectric behavior [51]. Through modulation, the Curie temperature of the material was considerably increased to 114.4 K, and a record-breaking emission lifetime of up to 117.11 ms was achieved under ambient temperature conditions. Therefore, this novel material may potentially be applied in multi-level density data storage, multifunctional sensors, and integrated optoelectronic devices with multitasking capabilities.

Compared with monatomic metal complexes, homopolymolybdate compounds possess a wide variety of structures due to the presence of a large number of molybdenum atoms and different coordination modes. Electron transfer between Mo and O atoms also confers good electrocatalytic properties [52,53,54,55,56,57,58,59]. In a study by Su et al. [60], 1,3-bis (1,2,4-triazol-1-yl)propane (btrp) and [*β*-Mo_8_O_26_]^4−^ were used as raw materials for the design and synthesis of [Co(btrp)_2_(H_2_O)_2_(*β*-Mo_8_O_26_)_0.5_]·H_2_O under hydrothermal conditions. The compound formed a two-dimensional (2D) mesh structure and exhibited good catalytic performance towards H_2_O_2_ and nitrosated amines, which was indicative of its excellent electrocatalytic functionality. Imidazole is an amphoteric organic compound having a five-membered aromatic heterocyclic diazole ring that contains two non-adjacent N atoms. The conjugation effect of the two N atoms makes them highly susceptible to protonation, which has led to the wide applicability of the imidazole ring in molecular design. For instance, Xiong et al. [61] designed and synthesized a pair of high-temperature organic enantiomeric ferroelectrics, imidazolium L-camphorsulfonate and imidazolium D-camphorsulfonate, using nonferroelectric imidazolium methanesulfonate as the raw material and adopting molecular symmetry-lowering design strategies. The two molecular-based functional materials exhibited good ferroelectric and phase transition properties.

**Figure 1:**
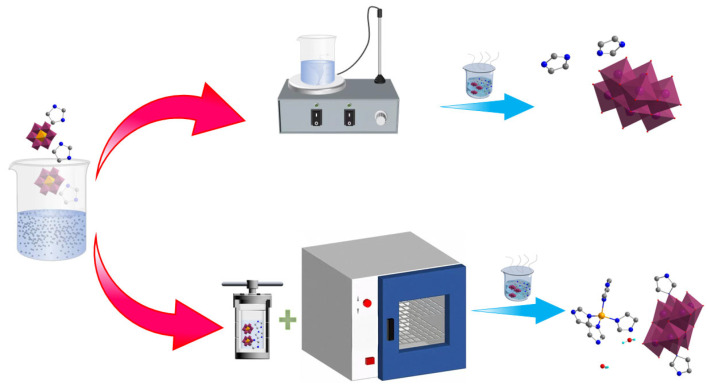
Schematic of compound synthesis pathways.

In the present study, the Anderson-type homopolyacid (NH_4_)_3_ [AlMo_6_O_24_H_6_]·7H_2_O, which has a known structure, and imidazole, which possesses multiple coordination sites, were used as raw materials for the design and synthesis of homopolyacid anionic compounds with small molecular volume (Figure 1). We report, for the first time, the adoption of a novel crystallization mode that involves the use of moderate-temperature hydrothermal and natural evaporation methods in a weakly acidic system for the successive promotion of crystal growth. Through metal ion modulation, different proportions of CaCl_2_·2H_2_O were introduced to synthesize organic–inorganic hybrid functional materials that contain *β*-type [Mo_8_O_26_] polyacid anions and a structure mainly characterized by free organic molecules. On this basis, Ca^2+^ ions were replaced by Zn^2+^ ions with smaller atomic radii to achieve the fixation of the free organic small molecules and promote the formation of coordination structures between metal ions and organic compounds. The Zn^2+^ ions readily coordinated with imidazole molecules to form fan-shaped metal–organic complexes, leading to the compression of the molecular spaces within the crystal structures. This induced the first reported formation of three-dimensional (3D) hydrogen-bonded imidazole-octamolybdenum-oxo cluster supramolecular materials containing the transitional *γ*-type [Mo_8_O_26_] polyacid anions. Subsequently, we determined the structures of the obtained materials and analyzed their dielectric and electrochemical performances. Results revealed that the structurally designed metal–organic complex-homopolyacid coordination compound {Zn(C_3_N_2_H_4_)_4_}{[(*γ*-Mo_8_O_26_)(C_3_N_2_H_4_)_2_]_0.5_}·2H_2_O (**2**) possessed an excellent 3D structure and exhibited a superior electrical performance. Therefore, the present study offers a novel approach for the synthesis of organic–inorganic hybrid functional homopolyacid compounds using heteropolyacids as raw materials.

## 2. Results and Discussion

### 2.1. Structural Analysis

Crystals that were crack-free and showed good crystal shapes were selected for single-crystal XRD analysis. The positions of atoms were determined using direct methods, and anisotropic refinement based on *F*^2^ was performed for non-hydrogen atoms using the SHELXL-97 program [62]. Table 1 shows the structural data and refinement parameters of **1** and **2**. Compounds **1** and **2** crystallized in the *P*-1 space group, with the molecular formulae of C_6_H_10_Mo_4_N_4_O_13_ and C_15_H_20_Mo_4_N_10_O_15_Zn, respectively. When the temperature was changed from 100 to 293 K, the following changes occurred in the unit cell parameters of **1** and **2**: *a* increased by 1.5% and 1.1%, respectively, *b* by 0.78% and 0.59, and *c* by 0.63% and 0.47%, whereas *α* decreased by 0.43% and 0.19%, *β* by 0.45% and 0.09%, and *γ* by 1.01% and 0.28%. During the heating process, obvious changes occurred in the *a*, *b*, *c*, and *γ* values in the unit cell of **1**, while **2** only showed a more significant change in the *a* value. This indicated that the unit cell structures of **1** and **2** possessed different sensitivities towards temperature changes, suggesting the presence of differences in the degree of structural change with temperature between the two compounds.

**Figure 2:**
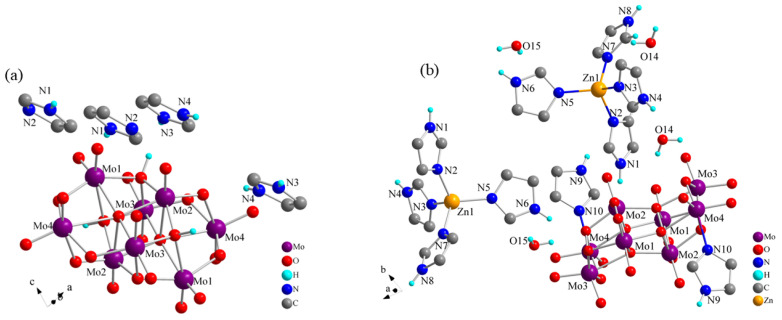
Simplest structures for symmetry operations of (**a**) **1** and (**b**) **2**.

Figure S1a,b show the crystal structures of **1** and **2**. Compound **1** contained half of an [*β*-Mo_8_O_26_H_2_] octamolybdenum-oxo cluster anion and two imidazoles (one imidazole molecule and one imidazole cation) (Figure S1a). The asymmetric unit of **2** consisted of half of an [(*γ*-Mo_8_O_26_)(C_3_N_2_H_4_)_2_] octamolybdenum-oxo cluster-imidazole complex anion, one [Zn (C_3_N_2_H_4_)_4_]^2+^ metal–organic complex cation, and two water molecules (Figure S1b). Figure 2 shows the simplest structures for symmetry operations of the two compounds (only certain H atoms are shown). In both **1** and **2**, the Mo and O atoms of the octamolybdenum-oxo cluster anion are bonded with six coordination bonds in an ortho-octahedral geometry. With high-pressure and high-temperature treatment during the synthesis of **2**, N atoms of the imidazole molecules replaced O atoms in the same position and formed coordination bonds with the Mo atoms. This resulted in self-organization of the two imidazole molecules and Mo atoms in the octamolybdenum-oxo cluster anion coordination structure of **2**, thereby forming the organic polyacid anionic group structure. Electrons of organic ligands could be readily accepted by the empty orbitals in the outermost layer of the Zn^2+^ ions. Under high-temperature and high-pressure conditions, four stable coordination bonds were formed among the N and Zn atoms of the four imidazole molecules, leading to the construction of a stable fan-like Zn atom coordination compound, as shown in Figure 2b. Compound **2** was obtained by the introduction of Zn atoms on the basis of **1**. Consequently, the [Zn(C_3_N_2_H_4_)_4_]^2+^ metal–organic complex cations formed by the Zn atoms and imidazole molecules led to changes in the structure of **2**. However, a comparison of the bond lengths and angles of the two compounds at different temperatures (Table S1) revealed that the imidazole cations or Zn complexes existed more stably in voids formed by the surrounding POMs, forming anion–cation hydrogen-bonded compounds with different structures.

**Figure 3:**
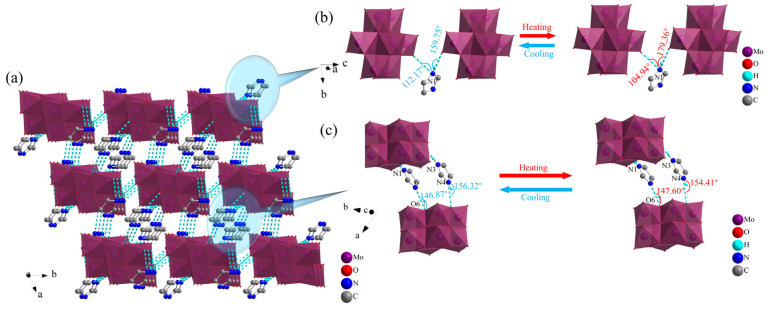
(**a**) Hydrogen bond stacking in **1**; Hydrogen bond changes of **1** along the (**b**) *c* axis and (**c**) *b* axis.

The simplest structure of **1** contained an independent imidazole cation and an octamolybdenum-oxo cluster anion. The imidazole cation served as a bridge along the *c* axis to form a one-dimensional (1D) chain-like structure through N–H···O hydrogen bonding interactions between the N atoms of the imidazole molecules and the terminal O atoms of two neighboring octamolybdenum-oxo cluster anions. The type of hydrogen bond in **1** was basically consistent with the hydrogen bond formed by the imidazolium-FCrO_3_ compound synthesized by Xiong et al. [41]. From the hydrogen bond data in Table S2, the bond angles of formed by the N1 atoms in the imidazole cations underwent significant changes during the transition from RT to LT, as shown in Figure 3b. The N1–H1···O1 and N1–H1···O2 bond angles were 104.94° and 179.36° at RT, respectively, and 112.17° and 159.75° at LT, with the changes being 6.89% and 12.28%. This indicated the occurrence of an obvious oscillation phenomenon in the anions and cations along the *c* axis, which induced changes in the physical properties of 1 with changes in temperature. By contrast, the O–H···N and N–H···O hydrogen bonding interactions between the imidazole cations and terminal O atoms of the octamolybdenum-oxo cluster anions along the *b* axis exhibited changes in their hydrogen bond angle of 0.49% and 1.23%, respectively, during the change from RT to LT. This demonstrated the occurrence of slight oscillations in the anions and cations along the *b* axis (Tables S2 and S3). These findings indicated that the anions and cations formed 1D chain-like structures along the *a*, *b*, and *c* axes via N–H···O and O–H···N hydrogen bonding interactions. As shown in Figure 3a, the [(*β*-Mo_8_O_26_H_2_) octamolybdenum-oxo cluster anions were linked to four surrounding imidazole molecules by hydrogen bonds, resulting in the formation of a hydrogen-bonded 3D structure in **1**.

**Figure 4:**
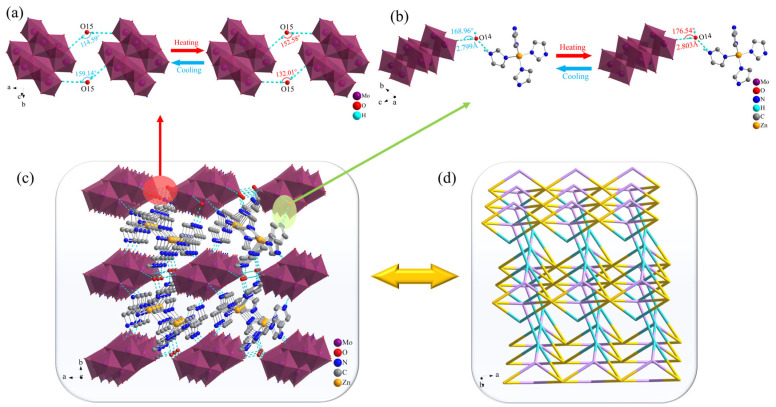
Hydrogen bond changes of **2** along the (**a**) *a* axis and (**b**) *c* axis; (**c**) Hydrogen bond stacking in **2**; (**d**) Topological representation of the stacking structure in **2**.

Compound **2** possessed a more complicated structure compared with **1**. As shown in Figure 4, the Mo atoms in the [(*γ*-Mo_8_O_26_)(C_3_N_2_H_4_)_2_] anion formed an octahedral structure with six surrounding O atoms. For better clarity in the stacking structure, imidazole molecules coordinated to the [(*γ*-Mo_8_O_26_)(C_3_N_2_H_4_)_2_] anion were omitted, with only the [*γ*-Mo_8_O_26_] structure that was similar to **1** retained in the diagram. O15 of the free water molecule served as a bridging link between the [Zn(C_3_N_2_H_4_)_4_]^2+^ metal–organic complex cation and the [(*γ*-Mo_8_O_26_)(C_3_N_2_H_4_)_2_] anion, forming a 1D chain-like structure along the *a* axis via O–H···O hydrogen bonding interactions with the terminal O atoms of two adjacent [(*γ*-Mo_8_O_26_)(C_3_N_2_H_4_)_2_] anions. As shown in Figure 4a, the bond angles of O15–H15A···O8 and O15–H15B···O11 between the O15 atom and [(*γ*-Mo_8_O_26_)(C_3_N_2_H_4_)_2_] anion changed from 152.08° and 132.01° to 114.59° and 159.14° at RT and LT, respectively, representing changes of 33.15% and 20.55%, respectively (Table S4). This indicted that obvious oscillations occurred in the 1D chain-like structures formed by the bridging effects of water molecules during the temperature change process, which induced certain physical property changes in the compound. The N atom of imidazole in the fan-like [Zn(C_3_N_2_H_4_)_4_]^2+^ metal–organic complex cation formed by coordination of Zn ions with imidazole readily formed N–H···O hydrogen bonds with terminal O atoms on the [(*γ*-Mo_8_O_26_)(C_3_N_2_H_4_)_2_] anion. The hydrogen bond type in **2** is similar to the [quinuclidinium] ReO_4_ crystal material synthesized by Tang et al. [42]. There were almost no differences in hydrogen bond lengths and angles of **2** along the *b* axis at different temperatures, indicating the absence of obvious oscillatory and stretching motion in the anions and cations in this direction (Figure S2). The O14–H14A···O12 hydrogen bonds formed by O14 atoms along the *c* axis changed by 0.14% and 4.49% with increasing temperature. This indicated the presence of slight stretching vibrations and more pronounced up-down oscillations in **2** in this direction, leading to differences in the physical properties of the compound. Another water molecule served as a bridge that linked the [(*γ*-Mo_8_O_26_)(C_3_N_2_H_4_)_2_] anion to the [Zn(C_3_N_2_H_4_)_4_]^2+^ metal–organic complex cation along the *c*-axis via O–H···O hydrogen bonding, jointly forming a 2D planar structure with the 1D chain-like structure along the *b* axis. To provide a clearer display of the 3D structural characteristics of **2**, the [(*γ*-Mo_8_O_26_)(C_3_N_2_H_4_)_2_] anion was simplified as a Mo atom, the Zn ion in the [Zn(C_3_N_2_H_4_)_4_]^2+^ metal–organic complex was retained, all other organic atoms were omitted, the H atoms of all water molecules in the structure were omitted, and all hydrogen bonds in **2** were denoted as solid lines and linked within the unit cell to obtain the 3D hydrogen-bonded stacking structure of **2** shown in Figure 4d.

### 2.2. Hirshfeld Surface Analysis

**Figure 5:**
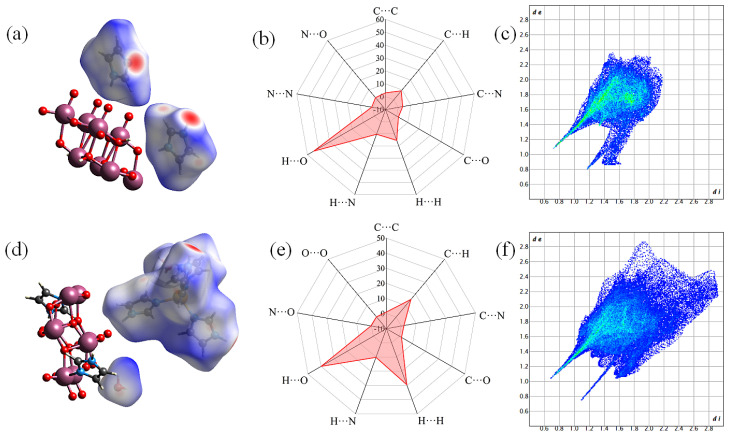
Hirshfeld surfaces of (**a**) **1** and (**d**) **2**; Radar charts of the contents of different hydrogen bonds in (**b**) **1** and (**e**) **2**; 2D fingerprint plots mapped with *d*_norm_ for (**c**) **1** and (**f**) **2**.

The large red spot in the Hirshfeld surface of **2** (Figure 5a) corresponded to the hydrogen bond formed between the uncoordinated N atom of the imidazole molecule in the [Zn(C_3_N_2_H_4_)_4_]^2+^ metal–organic complex and the O atom of a free water molecule, whereas the large red spots in the Hirshfeld surface of **1** (Figure 5d) represented the hydrogen bonds formed by the two free imidazole cations. A number of C atom-dominated hydrogen bonds were also present in certain regions of the Hirshfeld surface (Figure 5b,e). The fingerprint plots (Figure 5c,f) showed that O···H and H···O interactions accounted for 54.3% and 39.9% of the total interactions of the crystal in **1** and **2**, respectively. This suggests that N–H···O bonds were predominant and more abundant in **2** than in **1**. There was a greater presence of C···H, H···H, and C···O interactions within the unit cell of **2** than in **1**, and a greater presence of C···C, C···N, N···H, and N···O interactions in **1** than in **2** (Figure S3). Structural analysis revealed that O···O interactions were absent in **1** but accounted for 0.2% of the interactions in **2**. Such interactions arose from the presence of two water molecules in **2**, which caused the formation of O···O-type hydrogen bonds between the water molecules and the [(*γ*-Mo_8_O_26_)(C_3_N_2_H_4_)_2_] anion. This result was consistent with the presence of 1D hydrogen-bonded chain-like structures along the *a* axis in **2** (Figure S4). N···N interactions were also present with a contact ratio of 0.9% in **1**. This was mainly attributed to the small distance between the two imidazole molecules, with calculations revealing the presence of certain weak intermolecular forces. The Hirshfeld surface calculations demonstrated that different types of strong hydrogen bonding interactions existed within the crystal structures of **1** and **2**, contributing to the formation of mesh-like 3D hydrogen bonding patterns in the compounds.

### 2.3. XRD and Thermal Analysis

Figure S6 shows the X-ray powder diffraction patterns of **1** and **2** in the 2*θ* range of 10–50°, with patterns (ⅰ) and (ⅱ) corresponding to the measured and simulated diffraction peaks based on single-crystal XRD data, respectively. A comparison of peak values and positions of the two sets of data revealed an extremely high degree of similarity, demonstrating that both **1** and **2** were high-purity crystalline materials.

Figure S7 shows the results of thermal stability testing of the two compounds. The thermogravimetric analysis (TGA) curve showed that **1** started to decompose at 527 K. This indicated that **1** possessed good thermal stability. The differential thermal analysis (DTA) curve of **1** showed that the weight loss process of the compound was divided into three stages. During the first stage in the temperature range of 527–628 K, one imidazole molecule was lost, and the weight loss was approximately 9.8%, which is in agreement with the theoretical value of 9.3%. In the second stage at 628–724 K, the mass loss of another imidazole molecule was approximately 8.5%, which was also close to the theoretical value of 9.3%. The third stage occurred beyond 724 K. During this stage, the octamolybdenum-oxo cluster ionic skeleton of **1** underwent a gradual collapse until a stable oxide structure formed, after which decomposition ceased. The thermal decomposition process of **1** was consistent with its crystal structure and room-temperature infrared (IR) spectra.

Based on the DTA curve of **2**, it was preliminarily determined that thermal decomposition of the compound also occurred in three stages, with the middle two thermal peaks comprising one stage. The TGA curve indicated that **2** started to decompose at 331 K. During the first stage at 331–370 K, two water molecules were lost, with a weight loss of approximately 4.3%, which was consistent with the theoretical value of 3.5%. The second stage at 370–629 K involved a continuous decomposition process with a loss of four imidazole molecules. The actual weight loss was approximately 25.4%, which was in agreement with the theoretical value of 26.3%. The third stage occurred beyond 629 K. During this stage, the skeleton of the same polyanionic component of **2** underwent gradual collapse, and the remaining metal elements continued to exist as oxides with further increases in temperature. These results demonstrated that the thermal decomposition process of **2** was also consistent with its crystal structure.

### 2.4. Variable-Temperature Infrared (VTIR) Testing

**Figure 6:**
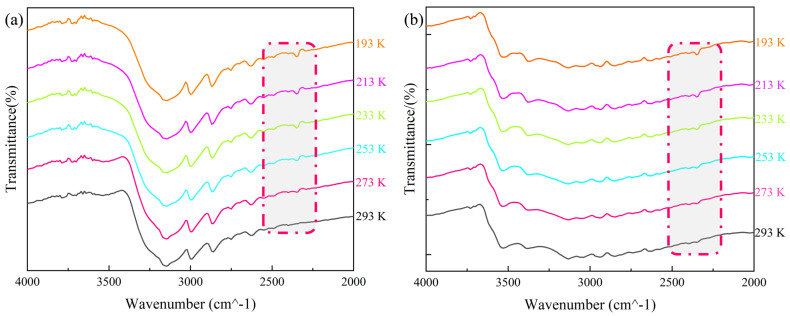
VTIR spectra for (**a**) **1** and (**b**) **2**.

In the IR spectrum of **1**, the peaks at 3138 and 1581 cm^−1^ were attributed to the stretching vibrations of unsaturated C–H in the imidazole molecules, and the stretching vibration of C=N, respectively, while the peaks at 942 and 906 cm^−1^ were the characteristic peaks of Mo–O in the *β*-Mo_8_O_26_H_4_ polyacid anion (Figure S4a). These results were consistent with the crystal structure of **1** and indicated the presence of two components in the compound, namely, imidazole molecules and [(*β*-Mo_8_O_26_H_2_)] anions. VTIR testing was carried out to further determine the structural changes of **1** with temperature. The VTIR spectra shown in Figure 6a indicate that within the range of 2200–2500 cm^−1^, **1** not only exhibited the original characteristic molecular peaks but also showed significant strengthening of the C–N–H characteristic peak at 2300 cm^−1^ when the temperature decreased from 293 to 193 K. With a gradual rise in temperature to approximately 273 K, characteristic peaks within this range were progressively weakened. The results corroborated the obvious change in the angle of the N–H···O hydrogen bond in **1** with a change in temperature, thereby demonstrating the presence of a relationship between N–H···O bond angle changes and characteristic peak weakening. This suggests that oscillations of the hydrogen-bonded structure formed by imidazole and the [(*β*-Mo_8_O_26_H_2_)] anion in **1** induced oscillations in the internal 3D structural framework, which induced thermal energy transfer within the structure.

Compared with the composition and spatial structure of **1**, **2** possessed substituted organic components and metal complexes. This led to a significant increase in the complexity of the IR spectrum of **2** (Figure S5b). Specifically, absorption peaks of water molecules appeared in the range of 3532–3391 cm^−1^, the stretching vibration peaks of C–H and C=N in the imidazole ring occurred at 3032 and 1548 cm^−1^, respectively, and the characteristic peaks of Mo–O and Zn–N were at 931 and 651 cm^−1^. The spectral results were consistent with the crystal structure of **2**, indicating the presence of imidazole molecules, polyacid anions, water molecules, and zinc complexes. Figure 6b shows the VTIR spectra of **2**. Within the range of 2212–2500 cm^−1^, an obvious characteristic peak was present at 193 K, but gradually weakened with an increase in temperature to 253 K. The weakening of the characteristic peak was caused by changes in the bond length and angle of the N–H···O hydrogen bond in **2**. This demonstrated that the stretching motion of the 3D hydrogen bonding mesh framework of **2** caused thermal energy transfer within the structure. This phenomenon is similar to the changes in the spectroscopic results of **1**, with obvious hydrogen bond characteristic peaks within the same wavenumber range disappearing and reappearing with changes in temperature. Therefore, it is evident that both hydrogen-bonded polyacid compounds were temperature-sensitive phase change materials.

### 2.5. Variable-Temperature Dielectric Testing

**Figure 7:**
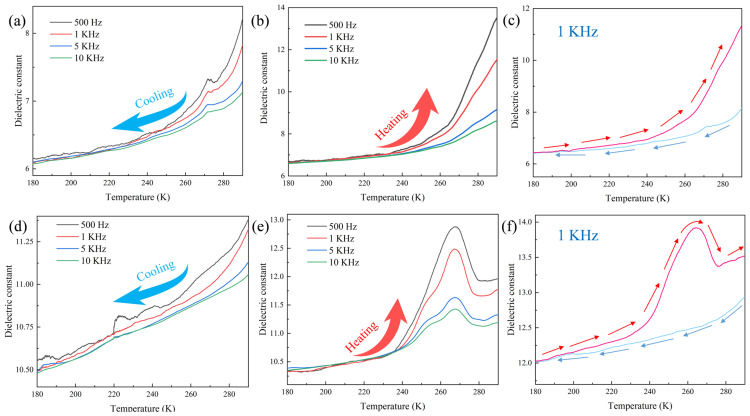
Dielectric constant curves of **1** during (**a**) cooling, (**b**) heating, and (**c**) cyclic testing at 1 kHz; Dielectric constant curves of **2** during (**d**) cooling, (**e**) heating, and (**f**) cyclic testing at 1 kHz.

The small crystal volumes of **1** and **2** posed difficulties for dielectric testing using the triaxial method. Therefore, dielectric measurements were performed on pellets. Figure 7a,b show that the dielectric constant of **1** in the frequency range of 500–10 kHz showed a negative correlation with frequency during the temperature change from 180 to 290 K. During the cooling process, the dielectric constant of **1** decreased with temperature in the temperature range of 290–160 K, with a small anomalous peak appearing at approximately 270 K. The dielectric constant gradually stabilized when temperature was decreased to approximately 200 K. The dielectric constant of **1** rose slowly at all frequencies within the range of 180–250 K. When the temperature was increased to 253 K, the dielectric constant showed a sharp increase, leading to the dielectric anomalies shown in Figure 7b. Based on the crystal structure, this phenomenon was primarily attributed to changes in the N–H···O bond angle within the multiaxial hydrogen-bonded chain-like structure in the material. Figure 7c shows the results of cyclic dielectric testing of **1** at 1 kHz, which indicated that the compound possessed good cyclicity. Testing performed at other frequencies also revealed the presence of good cyclicity, as shown in Figure S8.

Figure 7d,e show the results of dielectric testing of **2**. The trends of change of the dielectric constant of **2** with temperature were similar to those of **1**, with the dielectric constant being inversely proportional to frequency. During the cooling process, small peaks similar to those exhibited by **1** were not formed for **2**. This may be due to the gradual contraction of the unit cell volume of **2** during cooling, leading to a more regular pattern of change in the water molecules within its structure. Heating led to a sudden rapid increase in the dielectric constant of **2** when the temperature was increased beyond 240 K. The maximum values at the various frequencies were reached at approximately 265 K. Subsequently, the dielectric constant decreased within the temperature range of 265–280 K, and remained stable beyond 280 K. Therefore, the dielectric constant of **2** formed a distinct peak between 240 and 280 K. This phenomenon was attributed to the changes in the bond length and angle of the hydrogen bond between the two water molecules and the [(*γ*-Mo_8_O_26_)(C_3_N_2_H_4_)_2_] anion of the compound. Similar to **1**, **2** also exhibited good dielectric cyclicity at other frequencies, as shown in Figure S6.

These results demonstrated that the temperatures at which dielectric anomalies were produced are consistent with the temperatures at which anomalies were observed in the VTIR spectra. This suggests that changes in the bond angles of hydrogen bonds of different directions within the 3D hydrogen bonding structure had a high tendency of causing changes among the anions and cations and inducing changes in thermal and electric properties. Therefore, it is evident that both **1** and **2** could serve as novel dielectric anomaly-type crystalline materials. The dielectric properties of these two compounds are similar to those of [(CH_3_)_2_(F-CH_2_CH_2_)NH]_3_(CdCl_3_)(CdCl_4_) crystal synthesized by Wang et al. [43], indicating that these two compounds have good dielectric properties.

### 2.6. Electrochemical Testing

**Figure 8:**
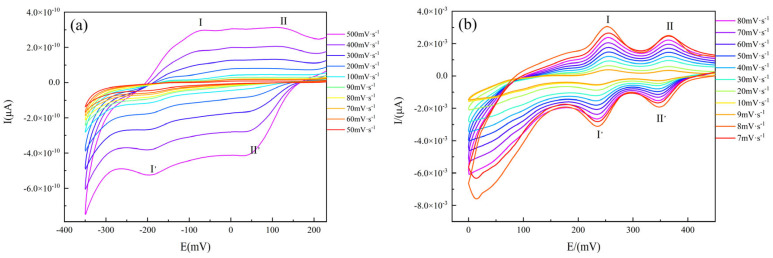
Cyclic voltammograms of (**a**) **1** and (**b**) **2**.

Figure 8 shows the results of cyclic voltammetry testing performed on glassy carbon electrodes prepared from **1** and **2**. The electrical performance of the compounds was tested at different scan rates, and the peak current (*i*_p_) was calculated using a combination of the Randleš–Sevčik and Nernst equations:

*i*_p_ = 2.69 × 10^5^*n*^3/2^*Ac*_o_*D*_o_^1/2^*v*^1/2^ (1)

where *A* is the electrode surface area (cm^2^), *v* is the scan rate (V s^−1^), *D*_o_ is the diffusion coefficient (cm^2^ s^−1^), and *c*_o_ is the bulk concentration of the electroactive substance (mol cm^−3^). When the ratio of the anodic and cathodic peak currents *i*_pa_/*i*_pc_ ≈ 1, the system was deemed to be reversible.

Compound **1** (Figure 8a) was a reversible system at scan rates below 200 V s^−1^, whereas the reductive current was greater than the oxidative current at scan rates above 200 V s^−1^. Compound **2** (Figure 8b) showed good reversibility over the entire scan rate range. The current of **2** was also considerably higher than that of **1**, which was attributed to the fact that the [(*γ*-Mo_8_O_26_)(C_3_N_2_H_4_)_2_] anion and the nearby fan-like [Zn(C_3_N_2_H_4_)_4_]^2+^ metal–organic complex cation of **2** formed the corresponding donor and acceptor for electron transfer. This led to the enhancement of electron transfer capacity between anions and cations. For **1**, two pairs of reversible redox peaks I-I’ and II-II’ existed at the scan rates of 50, 60, 70, 80, 90, 100, 200, 300, 400, and 500 mV s^−1^, with corresponding peak potentials *E*_1/2_ = (*E*_pa_ + *E*_pc_)/2 of −133 and +84 mV (scan rate: 500 mV s^−1^), respectively. The cyclic voltammetry characteristics of **2** were generally consistent with those of **1**. Two pairs of distinct reversible redox peaks I-I’ and II-II’ existed at the scan rates of 7, 8, 9, 10, 20, 30, 40, 50, 60, 70, and 80 mV s^−1^, with corresponding peak potential values of +245.5 and +357 mV (scan rate: 80 mV s^−1^), respectively. These results demonstrated that the electrochemical properties of the two compounds were similar to those of the molybdenum-oxygen homopolyacid organic–inorganic crystalline material synthesized by Wang et al. [63], suggesting that the redox processes of **1** and **2** were surface-controlled. The cyclic voltammograms also indicated that both compounds could form reversible electrochemical systems under the tested conditions.

### 2.7. Structural Void Calculations

**Figure 9:**
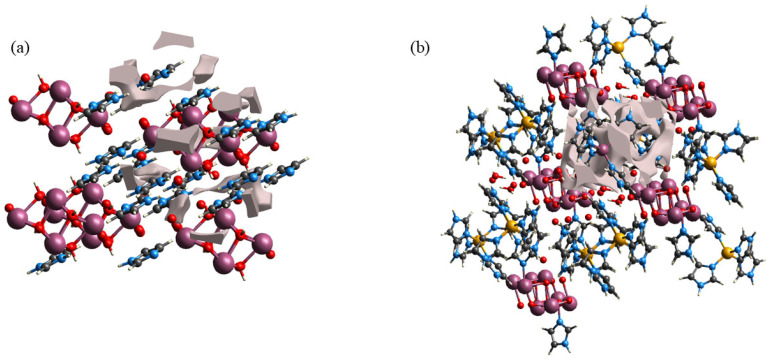
Void calculation results for (**a**) **1** and (**b**) **2**.

Calculations were performed for the hollow surfaces of the two compounds using the Surface Generation module of the CrystalExplorer software (21) to enable visualization of the empty regions in the crystal structure, and the results are shown in Figure 9. The void surfaces of the compounds were defined as the equivalent surfaces of the electron density of the original crystals and were calculated for the entire unit cell. When the surface of the void within the unit cell intersected with the boundary of the unit cell, a covering surface was generated to create a closed volume [64]. Figure S9 shows the resulting data of the spatial voids of the two compounds. The void volumes of **1** and **2** were 30.76 and 164.33 Å^3^, respectively, indicating that the void volume of **2** was more than 5 times that of **1**. Structural analysis of the two compounds revealed that the reason for the huge difference in void volume was the lack of strong rigid structures between the imidazole ion and [(*β*-Mo_8_O_26_H_2_)] anion of **1**. This led to compression of the space within the unit cell, resulting in a smaller void volume, and consequently, weakening the electron transfer ability. By contrast, **2** was composed of the [Zn(C_3_N_2_H_4_)_4_]^2+^ metal–organic complex cation, [(*γ*-Mo_8_O_26_)(C_3_N_2_H_4_)_2_], and water molecules. Therefore, a larger void volume existed within the unit cell, thus conferring a better electron transfer ability to the compound. These findings were in general agreement with the results of electrochemical testing, indicating that void volume exerted a considerable influence on electron transfer in these compounds.

### 2.8. Surface Electrostatic Potential Testing

**Figure 10:**
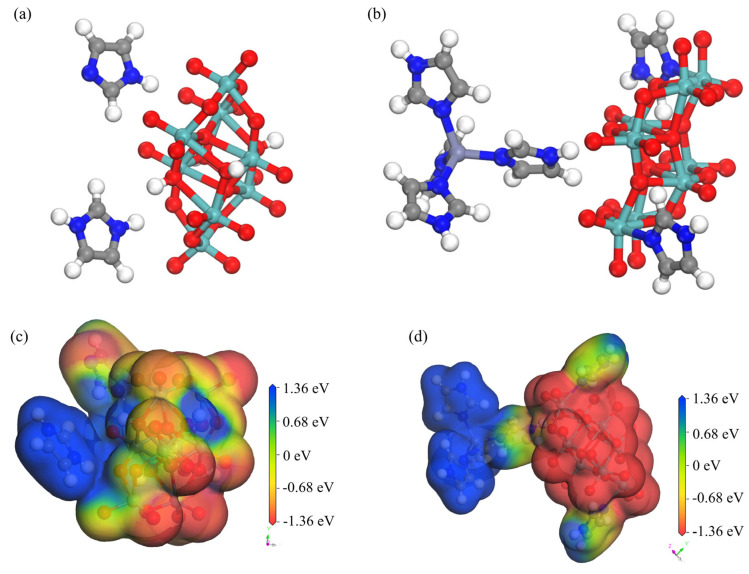
Simplified structures of (**a**) **1** and (**b**) **2**; simulated geometric shape and surface electrostatic potential of interactions of (**c**) **1** and (**d**) **2**.

Structural simulations were performed on the simplest structures for symmetry operation of **1** and **2** in Materials Studio software 2020(Figure 10a,b). The binding energy (*E*_bin_) between different molecules (*E*_bin_ = *E*_catalyst_ + *E*_species_ − *E*_system_) of **1** and **2** were determined to be 5.45 and 8.81 eV, respectively. Structural analysis of **1** and **2** revealed that the primary reason for the larger binding energy of **2** was the introduction of Zn^2+^ ions into its structure, thereby forming the bulkier [Zn(C_3_N_2_H_4_)_4_]^2+^ metal–organic complex cation. The fan-like structural characteristics of the metal complex led to a much larger anion—cation binding energy than that of **1**.

To investigate the electron transfer abilities of the two compounds, the density functional theory semi-core pseudopotential (DSPP) method was employed. During the optimization of structural geometry, the double numerical plus polarization (DNP) basis set was selected, and a certain degree of theoretical correction was applied to calculations for the introduction of transition metal atoms. The convergence thresholds for energy change, maximum force, and maximum displacement were set to 2 × 10^−5^ Ha, 0.004 Ha Å^−1^, and 0.005 Å, respectively. Figure 10c,d show a comparison of the active sites their surface electrostatic potentials. The surface electrostatic potentials around the polyacid anion and imidazole cation of **1** were approximately −1.36 and 1.36 eV, respectively [2]. However, due to the presence of strong π-bonds between the imidazole molecule and imidazole cation, there was an imbalance in electrons and molecular orbitals between neighboring imidazoles. This mainly contributed to a certain degree of irreversible cycling in **1** observed during cyclic voltammetry at high scan rates. Compound **2** differed from **1** in that its [Zn(C_3_N_2_H_4_)_4_]^2+^ metal–organic complex formed a more stable electron donor and acceptor pair with the [(*γ*-Mo_8_O_26_)(C_3_N_2_H_4_)_2_] homopolyacid complex. This led to the formation of a stable cyclic system, thus making **2** potentially applicable to the fields of electrocatalysis and energy storage.

## 3. Materials and Methods

### 3.1. Reagents and Instruments

Imidazole, ZnCl_2_, and HBF_4_ were purchased from Shanghai Macklin Biochemical Co., Ltd. (Shanghai, China). Ammonium molybdate, CaCl_2_·2H_2_O, and Al_2_(SO_4_)_3_ were purchased from Tianjin Zhiyuan Chemical Reagent Co., Ltd. (Tianjin, China). All reagents used were of analytical reagent grade.

IR and VTIR spectra were measured in the range of 400–4000 cm^−1^ using a Nicolet iS5 FT-IR Spectrometer (Thermo Scientific, Waltham, MA, USA). Crystal structures were determined using the same single crystal in a single-crystal X-ray diffractometer (Rigaku, Tokyo, Japan) with Mo-Kα radiation (λ = 0.71073 Å) under low-temperature (LT, 100 K) and room-temperature (RT, 293 K) conditions [65]. TGA was performed with a Q50 thermogravimetric analyzer (TA Instruments, New Castle, DE, USA), with Al_2_O_3_ used as the reference material under flowing nitrogen gas at a heating rate of 10 K min^−1^ and within the temperature range of 300–800 K. The temperature-dependent dielectric constants of the compounds were measured using a TH 2828 dielectric analyzer (Tonghui, Changzhou, China), with the pelletized test material coated on both sides with conductive silver paste and measurements performed in the frequency range of 1–100 kHz under an applied voltage of 1.0 V and temperature scan rate of 2 K min^−1^. The electrochemical performance of the compounds was tested using a CHI700E electrochemical workstation (Chenhua, Yangzhou, China), with the capacitance–voltage characteristic curves at different scan rates obtained by employing a three-electrode system in a mixed solution of 0.1 mol L^−1^ H_2_SO_4_ and 0.5 mol L^−1^ Na_2_SO_4_.

The Hirshfeld surface of the materials, commonly used for analyzing interactions among atoms within crystal structures, were analyzed using the CrystalExplorer program by utilizing principles of density functional theory (DFT) [66]. Structure simulation calculations were carried out using the DMol3 module in Materials Studio software (2020), with the Perdew–Burke–Ernzerhof functional adopted as the generalized gradient approximation method for calculating the exchange interactions.

### 3.2. Synthesis of Crystals

#### 3.2.1. Synthesis of [(C_3_N_2_H_5_)(C_3_N_2_H_4_)][(β-Mo_8_O_26_H_2_)]_0.5_ (**1**)

**Figure 11:**
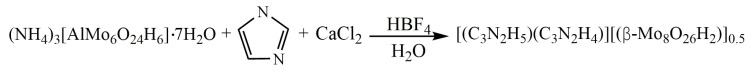
Synthetic route of compound **1**.

(NH_4_)_3_ [AlMo_6_O_24_H_6_]·7H_2_O [66], imidazole, and CaCl_2_·2H_2_O were used as raw materials for synthesis based on methods reported in the literature. Briefly, 0.20 g (0.17 mmol) (NH_4_)_3_[AlMo_6_O_24_H_6_]·7H_2_O, 0.09 g (1.37 mmol) imidazole, and 0.20 g (1.02 mmol) CaCl_2_·2H_2_O were weighed in accordance with the mole ratio of 1:8:6. The weighed raw materials were separately dissolved in 20.00 mL water, and the three solutions were uniformly mixed (Figure 11). One milliliter of a 45.00% HBF_4_ solution was added, and the mixed solution was thoroughly stirred and transferred to a round-bottomed flask. The contents of the flask were stirred and reacted at 80 °C for 0.5 h. After the reaction, the solution was filtered and the filtrate was transferred to a beaker, which was placed in a cool, windless place for slow evaporation. Seven days later, a block of colorless, transparent crystalline material was obtained from the bottom of the beaker. The yield was 26.45%.

#### 3.2.2. Synthesis of {Zn(C_3_N_2_H_4_)_4_}{[(γ-Mo_8_O_26_) (C_3_N_2_H_4_)_2_]_0.5_}·2H_2_O (**2**)

**Figure 12:**
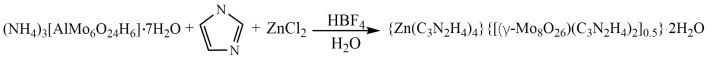
Synthetic route of compound **2**.

Compound **2** was synthesized using the same method and mole ratio as **1**, except that CaCl_2_·2H_2_O was replaced by ZnCl_2_. Briefly, 0.20 g (0.17 mmol) (NH_4_)_3_[AlMo_6_O_24_H_6_]·7H_2_O, 0.09 g (1.37 mmol) imidazole, and 0.20 g (1.02 mmol) ZnCl_2_ were weighed and separately dissolved in 15.00 mL water. After mixing the solutions, 1.00 mL of a 45.00% HBF_4_ solution was added, and the mixed solution was loaded into a polytetrafluoroethylene liner for reaction in a high-temperature autoclave at 120 °C for 24 h (Figure 12). The resulting solution was cooled, transferred to a beaker, and placed in a cool, windless place. Approximately one week later, a block of colorless, transparent crystalline material was obtained. The yield was 30.21%.

#### 3.2.3. Preparation of Glassy Carbon Electrodes

The test compound and carbon black were placed in a mortar at a mass ratio of 1:5, thoroughly ground, and dissolved in a mixed water and ethanol solvent. After uniform mixing, 5.00 μmL of the solution was evenly spread onto the surface of a glassy carbon electrode. A certain amount of naphthol solution was added dropwise and allowed to dry into a film before the electrode was used for testing.

## 4. Conclusions

Herein, the metal element substitution approach was adopted for the design and synthesis of two novel octamolybdenum-oxo cluster organic–inorganic hybrid compounds, [(C_3_N_2_H_5_)(C_3_N_2_H_4_)][(*β*-Mo_8_O_26_H_2_)]_0.5_ (**1**) and {Zn(C_3_N_2_H_4_)_4_}{[(*γ*-Mo_8_O_26_)(C_3_N_2_H_4_)_2_]_0.5_}·2H_2_O (**2**). The structures and properties of the obtained compounds were characterized by single-crystal XRD, VTIR, variable-temperature dielectric testing, and electrochemical testing. In **1**, the presence of imidazole molecules in the free state induced the formation of the β-type anionic structure by the molybdenum homopolyacid. By contrast, metal ion substitution in **2** induced the formation of the rarely reported *γ*-type anionic structure, and the combination of imidazole molecules in the free state with Zn ions led to the formation of a composite material consisting of a homopolyacid complex and a metal–organic complex. Structural analysis revealed that the 3D structures of **1** and **2** were formed through intermolecular hydrogen bonding. Multidimensional hydrogen bonding analysis indicated that the imidazole cation in **1** exhibited molecular oscillations between the [(*β*-Mo_8_O_26_H_2_)] anions, and that the water molecules between the [(*γ*-Mo_8_O_26_)(C_3_N_2_H_4_)_2_] anions in **2** showed a similar oscillation phenomenon through hydrogen bond interactions. Anomalous peak values during VTIR and dielectric anomalies of the compounds occurred at temperatures of approximately 250 and 260 K, respectively. The consistency in temperature ranges demonstrated that the obvious dielectric anomalies in both compounds under variable temperature conditions were mainly attributed to changes in bond angles of multidimensional hydrogen bonds. The results of simulation of the hollow surfaces and surface electrostatic potential of the compounds were consistent with the results of electrochemical testing. This indicated that the anions and cations constructed via hydrogen bonding interactions possessed good electron transfer ability. Our results have demonstrated the immense application potential of these two novel types of homopolyacids and homopolyacid-metal complexes in the fields of dielectric switching and electrocatalysis.

## Figures and Tables

**Table 1:** Crystallographic data of **1** and **2** at LT and RT.

	1		2	
Temperature/K	100 K	293 K	100 K	293 K
Chemical formula	C_6_H_10_Mo_4_N_4_O_13_	C_6_H_10_Mo_4_N_4_O_13_	C_15_H_20_Mo_4_N_10_O_15_Zn	C_15_H_20_Mo_4_N_10_O_15_Zn
Formula weight	730.95	730.95	1033.57	1033.57
Crystal size (mm^3^)	0.12 × 0.1 × 0.08	0.12 × 0.1 × 0.08	0.13 × 0.12 × 0.1	0.13 × 0.12 × 0.11
Crystal system	Triclinic	Triclinic	Triclinic	Triclinic
Space group	*P*-1	*P*-1	*P*-1	*P*-1
*a*/Å	9.209 (7)	9.348 (5)	10.511 (6)	10.626 (4)
*b/*Å	9.681 (7)	9.756 (7)	10.758 (4)	10.822 (3)
*c/*Å	10.301 (5)	10.367 (5)	14.512 (8)	14.581 (6)
*α/*(°)	84.321 (5)	83.982 (5)	69.058 (4)	68.926 (3)
*β/*(°)	76.044 (5)	75.700 (4)	83.033 (5)	83.110 (3)
*γ/*(°)	65.536 (7)	64.871 (6)	85.854 (4)	86.091 (3)
*V/*(Å^3^)	811.23 (11)	829.42 (9)	1520.45 (14)	1552.56 (10)
*Z*	2	2	2	2
*D*_c/_(g cm^−3^)	2.992	2.927	2.258	2.211
*F*(000)	694	694	1004	1004
*μ*/mm^−1^	3.101	3.033	2.464	2.413
2*θ* range/(°)	0.845–25.242	0.853–25.242	0.999–24.997	0.999–25.000
*R*_int_	0.034	0.031	0.059	0.029
*R*_1_ [*I* > 2σ(*I*)] ^a^	0.046	0.037	0.067	0.035
*wR*_2_ (all data) ^b^	0.084	0.077	0.154	0.066
GOF	1.038	1.048	1.031	1.029
CCDC	2,449,483	2,449,484	2,449,506	2,449,507

^a^ *R*_1_ = ∑(|*F*_o_| − |*F*_c_|)/∑|*F*_o_|; ^b^ *wR*_2_ = [∑*w* (|*F*_o_|^2^ − |*F*_c_|^2^)^2^/∑*w*(*F*_o_^2^)]^1/2^.

## Data Availability

Data can be provided upon reasonable request from the corresponding author.

## Supplementary Materials

The following supporting information can be downloaded at: https://www.mdpi.com/article/10.3390/molecules30153107/s1. Table S1: The main bond length and bond angle of compound **1**; Table S2: The main bond length and bond angle of compound **2**; Table S3: The bond length and bond angle data of hydrogen bonds in compound **1**; Table S4: The hydrogen bond table of compound **2**; Figure S1: (a) Compound **1** and (b) The simplest composition diagram of compound **2**; Figure S2: Compound **2** bc 2D hydrogen bond plane diagram; Figure S3: (a–k) The 2D fingerprint of compounds **1** with dnorm as parameter was formed; Figure S4: (a–l) The 2D fingerprint of compounds **2** with dnorm as parameter was formed; Figure S5: (a) Compound **1** and (b) Compound **2** IR spectra; Figure S6: XRD spectra of (a) **1** and (b) **2**; Figure S7: (a) Compound **1** and (b) Compound **2** TG test diagram; Figure S8: Compounds **1** 500 Hz (a), 5 KHz (b), 10 KHz (c) cyclic dielectric; compounds **2** 500 Hz (d), 5 KHz (e), 10 KHz (f) cyclic dielectric; Figure S9: (a) Compound **1**, (b) compound **2**, hole calculation diagram. Figure S10. The cyclic test of compound **1** at 100 (a) and 200 ms (b), and the cyclic test of compound **2** at 6 (c) and 7 ms (d).

## Abbreviations

The following abbreviations are used in this manuscript:

DFT	Density functional theory
DSPP	DFT semi-core pseudopotentials
DTA	Differential thermal analysis
DNP	Double numerical plus polarization
POM	Polyoxometalate
IR	Room-temperature infrared
TGA	Thermogravimetric analysis
VTIR	Variable-temperature infrared
XRD	X-ray diffraction
