# Supplementary material for: Design, Synthesis, and Electrical Performance of Three-Dimensional Hydrogen-Bonded Imidazole-Octamolybdenum-Oxo Cluster Supramolecular Materials
Source: Molecules. 2025 Jul 24;30(15):3107. doi: 10.3390/molecules30153107 (PMC12348099; doi:10.3390/molecules30153107)
Supplement: Supplementary file 1 [file molecules-30-03107-s001.zip › molecules-3769308-supplementary.pdf]

Design, Synthesis, and Electrical Performance of Three-Dimensional Hydrogen-Bonded Imidazole-Octamolybdenum-Oxo Cluster Supramolecular Materials

Hongzhi Hu [1,2], Adila Abuduheni [1], Yujin Zhao [1], Yuhao Lin [1], Yang Liu [1,2,3,*] and Zunqi Liu [1,2,3,*]

1. Chemistry and Chemical Engineering College, Xinjiang Agricultural University, Urumqi 830052, China; huhongzhi305@163.com (H.H.); 17799751675@163.com (A.A.); 18599215733@163.com (Y.Z.); lin18030922395@163.com (Y.L.)
2. Xinjiang Sub-Center National Engineering Research Center of Novel Equipment for Polymer Processing, Urumqi 830052, China
3. Xinjiang Key Laboratory of Agricultural Chemistry and Biomaterials, Urumqi 830052, China

* Correspondence: ly2021@xjau.edu.cn (Y.L.); lzq@xjau.edu.cn (Z.L.)

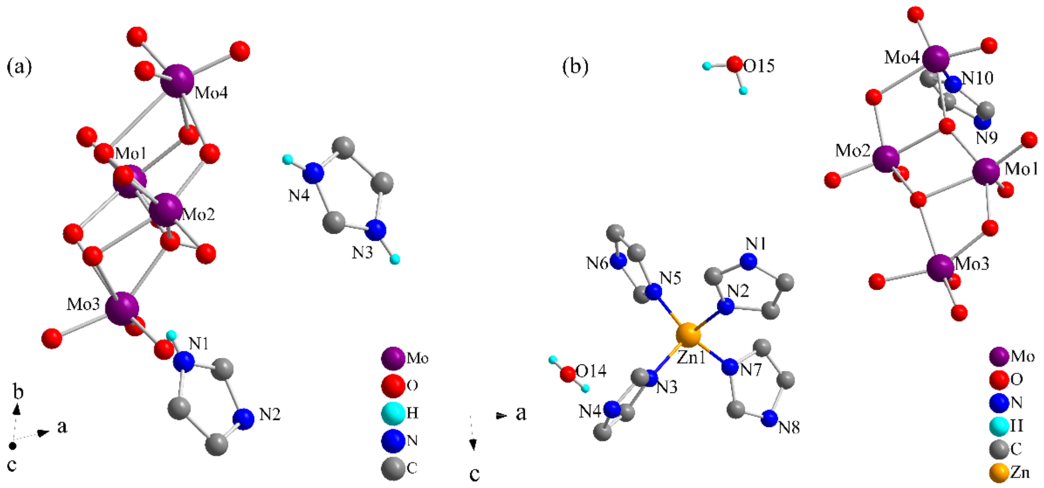

Figure S1 The simplest composition diagram of (a) compound **1** and (b) compound **2**

The X-ray single crystal diffraction test of compound **1** can obtain the simplest component diagram of the symmetrical operation of the polyoxoanions shown in Fig.S1 (a) (only part of the hydrogen atoms are shown in the figure), which contains 0.5 [β-$Mo_8O_{26}H_4$] polyoxoanions and two imidazole molecules. The X-ray single crystal diffraction test of compound **2** can obtain the simplest composition diagram shown in figure S1 (b). The minimum asymmetric unit is composed of 0.5 [(β-Mo_8O_{26})($C_3N_2H_4$)$_2$] polyoxometalate organic complexes, a $Zn(C_3N_2H_4)_4$ organic metal complex and two molecules of water.

Table S1 The main bond length and bond angle of compound **1**

Bond	Bond Length / Å	Bond	Bond Length / Å	Bond	Bond Angle (°)
		100K			
Mo1-O1	1.697(3)	Mo3-O3	2.349(3)	O1-Mo1-O2	105.80(16)
Mo1-O2	1.700(3)	Mo3-O6	2.030(3)	O1-Mo1-O3	101.12(15)
Mo1-O3	1.991(3)	Mo3-O7	1.702(4)	O1-Mo1-O6	88.23(13)
Mo1-O6	2.333(3)	Mo3-O9	1.889(3)	O1-Mo1-O8	100.11(16)
Mo1-O8	1.903(3)	Mo3-O12	2.283(3)	O1-Mo1-O12	160.56(13)
Mo1-O12	2.324(3)	Mo3-O13	1.706(3)	O2-Mo1-O3	97.31(15)
Mo2-O3	1.928(3)	Mo4-O5	2.277(3)	O2-Mo1-O6	163.78(15)
Mo2-O4	1.687(3)	Mo4-O8	1.914(3)	O2-Mo1-O8	101.59(15)
Mo2-O5	1.769(3)	Mo4-O9	1.916(3)	O2-Mo1-O12	93.43(14)
Mo2-O6	1.963(3)	Mo4-O10	1.694(4)	O3-Mo1-O6	71.52(12)
Mo2-O12	2.384(3)	Mo4-O11	1.712(3)	O3-Mo1-O12	73.01(12)
		293K			
Mo1-O1	1.697(2)	Mo3-O3	2.365(2)	O1-Mo1-O2	105.81(13)
Mo1-O2	1.705(3)	Mo3-O6	2.026(3)	O1-Mo1-O3	101.32(12)
Mo1-O3	1.994(2)	Mo3-O7	1.691(3)	O1-Mo1-O6	87.85(11)
Mo1-O6	2.350(2)	Mo3-O9	1.896(3)	O1-Mo1-O8	99.83(13)
Mo1-O8	1.897(3)	Mo3-O12	2.295(2)	O1-Mo1-O12	160.05(11)
Mo1-O12	2.311(2)	Mo3-O13	1.708(3)	O2-Mo1-O3	97.58(12)
Mo2-O3	1.922(2)	Mo4-O5	2.290(2)	O2-Mo1-O6	164.16(10)
Mo2-O4	1.686(3)	Mo4-O8	1.918(3)	O2-Mo1-O8	101.52(13)
Mo2-O5	1.761(3)	Mo4-O9	1.912(2)	O2-Mo1-O12	93.98(11)
Mo2-O6	1.957(2)	Mo4-O10	1.695(3)	O3-Mo1-O6	71.39(9)
Mo2-O12	2.393(2)	Mo4-O11	1.707(3)	O3-Mo1-O12	72.99(9)
Mo2-O12	2.132(2)	Mo3-O3	2.365(2)	O8-Mo1-O3	146.30(10)

Through the data of table S1, it was found that there was no obvious structural change in the homopolyacid structure of compound **1**, and the imidazole molecule was also stable in the crystal cell.

Table S2 The main bond length and bond angle of compound **2**

Bond	Bond Length / Å	Bond	Bond Length / Å	Bond	Bond Angle (°)
		100K			
Mo1-O1	1.712(5)	O1-Mo1-O2	103.2(2)	O0D-Mo3-O9	104.7(2)
Mo1-O2	1.719(4)	O1-Mo1-O3	100.5(2)	O0D-Mo3-O10	97.7(2)
Mo1-O3	1.922(4)	O1-Mo1-O6	83.67(19)	O5-Mo3-Mo4	43.95(12)
Mo1-O6	2.361(5)	O1-Mo1-O7	155.42(19)	O7-Mo3-Mo4	85.87(12)
Mo1-O7	2.209(5)	O1-Mo1-O8	105.9(2)	O7-Mo3-O5	73.54(15)

Mo1-O8	1.927(4)	O2-Mo1-O3	98.42(19)	O8-Mo3-Mo4	127.19(13)
Mo2-O3	2.151(5)	O2-Mo1-O6	172.93(19)	O8-Mo3-O5	83.38(18)
Mo2-O4	1.685(4)	O2-Mo1-O7	101.2(2)	O8-Mo3-O7	73.09(17)
Mo2-O5	1.912(4)	O2-Mo1-O8	96.96(19)	O9-Mo3-Mo4	87.71(16)
Mo2-O6	1.752(5)	O3-Mo1-O6	81.92(16)	O9-Mo3-O5	89.47(19)
Mo2-O7	2.471(4)	O3-Mo1-O7	72.69(18)	O9-Mo3-O7	161.0(2)
Mo2-O7	1.945(4)	O3-Mo1-O8	145.3(2)	O9-Mo3-O8	96.9(2)
Mo3-Mo4	3.2082(8)	O7-Mo1-O6	72.08(16)	O9-Mo3-O10	100.0(2)
Mo3-O00D	1.697(5)	O8-Mo1-O6	79.18(17)	O10-Mo3-Mo4	32.37(13)
Mo3-O5	2.294(5)	O8-Mo1-O7	73.92(17)	O10-Mo3-O5	74.35(18)
			293K		
Mo1-O1	1.717(2)	O1-Mo1-O3	101.07(12)	O0D-Mo3-O9	104.75(13)
Mo1-O2	1.711(2)	O1-Mo1-O6	83.43(10)	O0D-Mo3-O10	97.39(11)
Mo1-O3	1.925(2)	O1-Mo1-O7	154.78(10)	O7-Mo3-O5	73.51(8)
Mo1-O6	2.373(2)	O1-Mo1-O8	105.63(12)	O8-Mo3-O5	83.48(9)
Mo1-O7	2.215(2)	O2-Mo1-O1	103.45(12)	O8-Mo3-O7	73.15(9)
Mo1-O8	1.935(3)	O2-Mo1-O3	98.02(12)	O9-Mo3-O5	89.38(11)
Mo2-O3	2.150(2)	O2-Mo1-O6	172.96(10)	O9-Mo3-O7	160.70(11)
Mo2-O4	1.694(2)	O2-Mo1-O7	101.58(10)	O9-Mo3-O8	96.52(12)
Mo2-O5	1.912(3)	O2-Mo1-O8	97.33(12)	O9-Mo3-O10	100.19(12)
Mo2-O6	1.749(2)	O3-Mo1-O6	81.90(9)	O10-Mo3-O5	74.33(9)
Mo2-O7	2.480(2)	O3-Mo1-O7	72.22(9)	O10-Mo3-O7	84.01(10)
Mo2-O7	1.945(2)	O3-Mo1-O8	145.01(10)	O10-Mo3-O8	151.90(11)
Mo3-O00D	1.701(2)	O7-Mo1-O6	71.64(8)	O3-Mo4-O5	69.73(9)
Mo3-O5	2.303(2)	O8-Mo1-O6	79.16(10)	O3-Mo4-N10	79.00(11)
Mo3-O7	2.237(2)	O8-Mo1-O7	73.99(9)	O10-Mo4-O3	90.36(10)

As the temperature increases, the oxygen atom occupancy of compound **2** also increases, and the bond length and bond angle in the polyoxoanion skeleton does not change significantly as shown in Table S2.

Table S3 The bond length and bond angle data of hydrogen bonds in compound **1**

D-H···A	d(D-H) Å	d(H-A) Å	d(D-A) Å	D-H···A (°)
		100K		
N1-H1···O2	0.713	2.154	2.833	159.75
N1-H1···O1	0.713	2.711	3.052	112.17
N3-H3A···O11	0.860	1.985	2.840	172.88
N3-H3A···O7	0.860	2.689	3.014	103.96
N4-H4···O5	0.802	2.022	2.776	156.32
O6-H6···N2	0.980	1.956	2.828	146.87
		293K		
N1-H1···O2	0.797	2.035	2.832	179.36
N1-H1···O1	0.797	2,810	3.112	104.94

N3-H3A···O11	0.860	2.026	2.876	169.65
N3-H3A···O7	0.860	2.726	3.053	104.21
N4-H4···O5	0.798	2.074	2.814	154.41
O6-H6···N2	0.980	1.989	2.865	147.60

From the data in table S3, it can be seen that during the transition from room temperature to low temperature, the two hydrogen bond angles of N3-H3A···O11 and N3-H3A···O7 formed by the nitrogen atom in the imidazole molecule and the terminal oxygen atom of the same polyoxoanion changed from 169.65 ° and 104.21 ° (RT) to 172.88 ° and 103.96 ° (LT). With the decrease of temperature, the compound has a slight swing phenomenon, which leads to the change of physical properties of the compound **1**.

Table S4 The hydrogen bond of compound **2**

D-H···A	d(D-H) Å	d(H-A) Å	d(D-A) Å	D-H···A (°)
		100K		
N1-H1···O1	0.860	1.871	2.721	169.84
N8-H8···O15	0.860	1.951	2.782	162.21
N6-H6···O5	0.860	1.939	2.770	162.23
N4-H4···O14	0.860	1.961	2.778	158.51
O15-H15A···O8	0.845	2.297	2.758	114.59
O15-H15B···O11	0.842	2.059	2.862	159.14
O14-H14A···O12	0.845	1.964	2.799	168.96
		293K		
N1-H1···O1	0.861	1.874	2.724	169.19
N8-H8···O15	0.860	1.989	2.822	162.75
N6-H6···O5	0.859	1.975	2.799	160.40
N4-H4···O14	0.860	1.980	2.799	158.71
O15-H15A···O8	0.849	2.014	2.795	152.58
O15-H15B···O11	0.850	2.252	2.891	132.01
O14-H14A···O12	0.849	1.954	2.803	176.54

According to the hydrogen bond data in table S4, the average bond length and average bond angle of N-H···O formed at 100 K are 2.746 Å and 166.04 °, respectively. At 293 K, the average bond length and average bond angle are 2.7615 Å and 164.79 °, respectively. Compared with the above data, it is found that compound **2** has almost no obvious stretching vibration in the b-axis direction, but has a slight swing phenomenon. This shows that the change of the bond angle of compound **2** in the *b*-axis direction will also lead to a corresponding change in the structure of the compound.

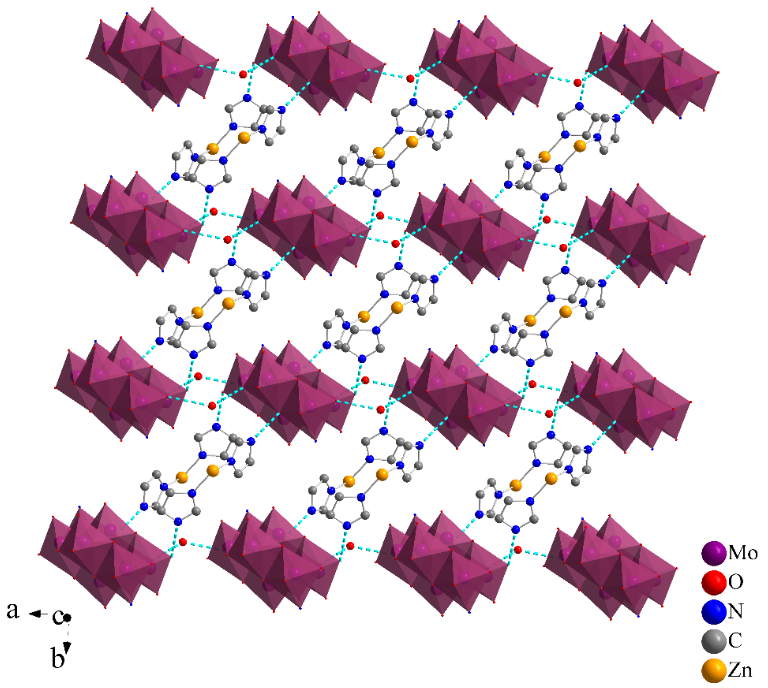

Figure S2 2D hydrogen bond plane diagram of compound **2** along the *bc* axis

The N atoms of imidazole in the 'fan' -like organic complexes formed by the coordination of imidazole and Zn ions are easily combined with the terminal oxygen atoms on the same polyanion in the form of N-H···O hydrogen bonds to form a one-dimensional structure in the b-axis direction at the periphery of the ' fan ' blades, so a stable two-dimensional network structure is formed in the ab plane as shown in Fig.S2.

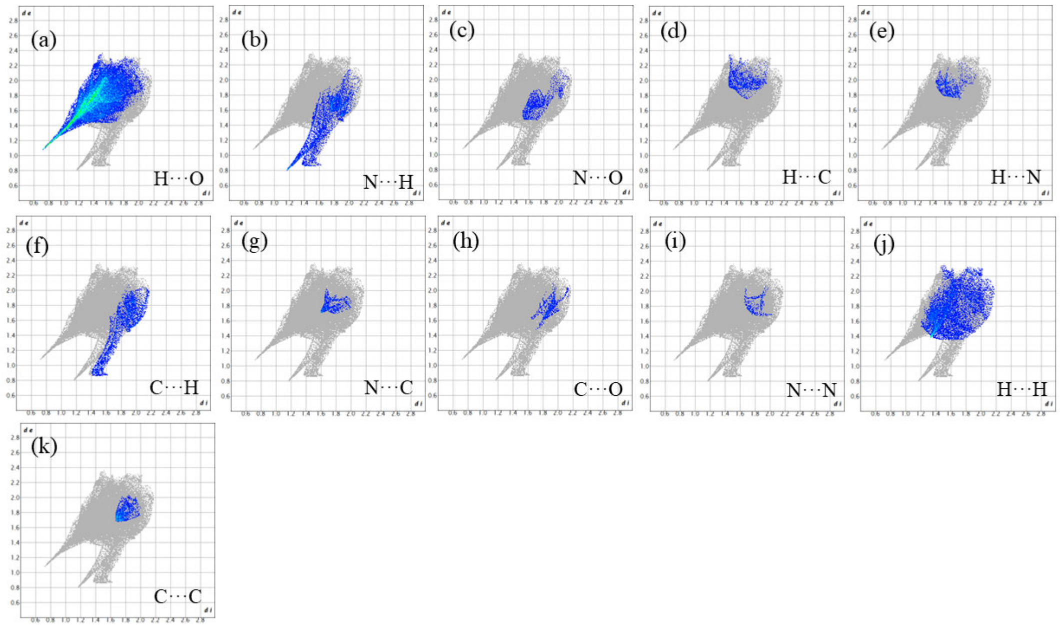

Figure S3 (a-k) The 2D fingerprint of compound **1** with dnorm as parameter was formed

In the Hirshfeld surface of compound **1**, the larger red spots correspond to the hydrogen bonds formed by the water molecules and the uncoordinated N atoms on the cation imidazole of the formed Zn metal complex (Fig.S3 (a)). The larger red spots in the Hirshfeld surface of compound **1** are two free imidazole molecules. There are also some hydrogen bonds with C atoms as the main body in some regions of the Hirshfeld surface. It can be seen from the fingerprint that the contact types of O···H and H···O in compound 1 account for 54.3 % of the whole crystal. The contact effects between C··· H, H···H and C···O in the cell of compound 1 are 9 %, 15.4 % and 1.5 %, respectively. In addition, the contact effects of C···C, C···N, N···H and N···O in compound 1 are 3 %, 3.5 %, 10.3 % and 2.1 %, respectively. Compound **1** also has a N···N-type contact effect alone, and its contact ratio is 0.9 %. This is due to the small distance between the two imidazole molecules.

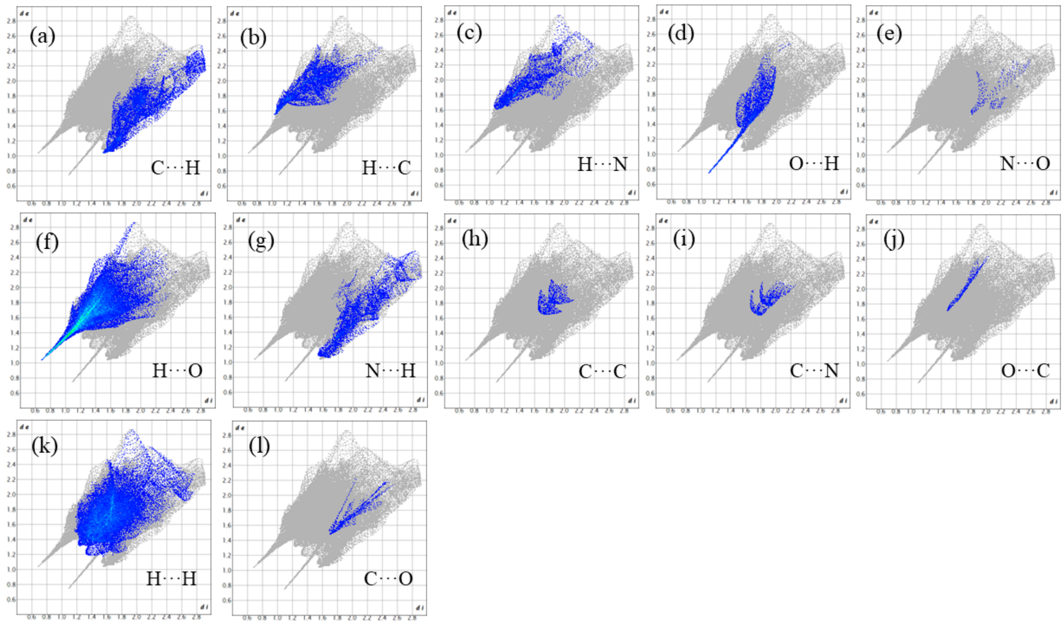

Figure S4 (a-l) The 2D fingerprint of compound 2 with dnorm as parameter was formed

On the Hirshfeld surface of compound 2, hydrogen bonds with O atoms as the main body exist in some regions of the Hirshfeld surface. It can be seen from the fingerprint that the contact type of O···H and H···O in compound 2 accounts for 39.9% of the entire crystal. The intracellular C··· H, H···H and C···O contact effects of compound 2 were 15.3%, 29.5% and 1.6%, respectively. In addition, the contact effects of C···C, C···N, N···H and N···O in compound 2 were 1%, 1.8%, 10.2% and 0.4%, respectively.

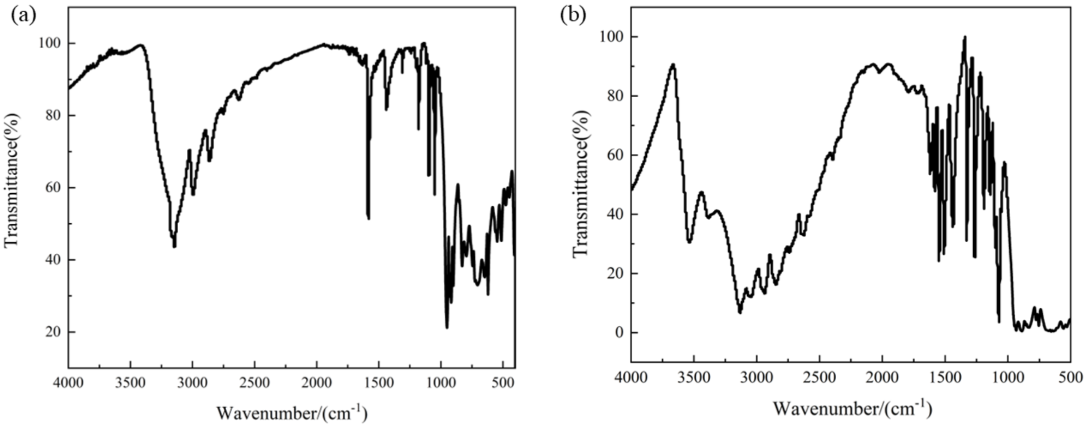

Figure S5 IR spectra of (a) compound **1** (b) compound **2**

The complete crystal material was selected and mixed with dried KBr to make a sample sheet. The infrared test results of compound **1** in the wavelength range of 400 cm^{-1}-4000 cm^{-1} are shown in Fig.S5 (a). At 3138 cm^{-1} is the stretching vibration of unsaturated C-H in imidazole molecule; at 1581 cm^{-1} is the stretching vibration of C = N; the characteristic peaks of Mo-O in β-$Mo_8O_{26}H_4$ polyoxoanion are at 942 cm^{-1} and 906 cm^{-1}, respectively. It can be preliminarily judged that compound **1** contains imidazole molecule and β-$Mo_8O_{26}H_4$ polyacid.

The compound **2** with relatively complete crystal form was selected and mixed with KBr crystal to make a sample sheet. The infrared test results in the wavelength range of 400-4000 cm^{-1} are shown in Fig.S5 (b). The characteristic peak at 3532 cm^{-1}-3391 cm^{-1} is the absorption peak of water molecules. The characteristic peak at 3032 cm^{-1} is the stretching vibration peak of C-H on the imidazole ring. The characteristic peak at 1548 cm^{-1} is the stretching vibration peak of C = N in imidazole ring. The characteristic peak at 931 cm^{-1} is the characteristic peak of Mo-O. The characteristic peak at 651 cm^{-1} is the characteristic peak of Zn-N. Therefore, it can be preliminarily judged from the infrared spectrum of compound **2** that compound **2** contains complexes of imidazole molecules, polyoxoanions, water molecules and Zn ions with imidazole molecules.

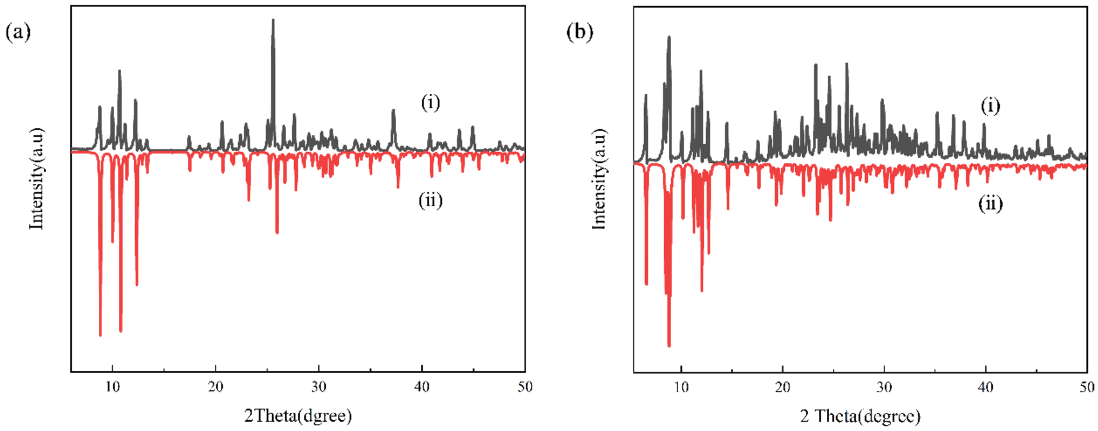

Figure S6. XRD spectra of (a) compound 1 and (b) compound 2.

Figure S6 shows the X-ray powder diffraction patterns of 1 and 2 in the 2θ range of 10 – 50°, with patterns (i) and (ii) corresponding to the measured and simulated dif-fraction peaks based on single-crystal XRD data, respectively. A comparison of peak values and positions of the two sets of data revealed an extremely high degree of simi-larity, demonstrating that both 1 and 2 were high-purity crystalline materials.

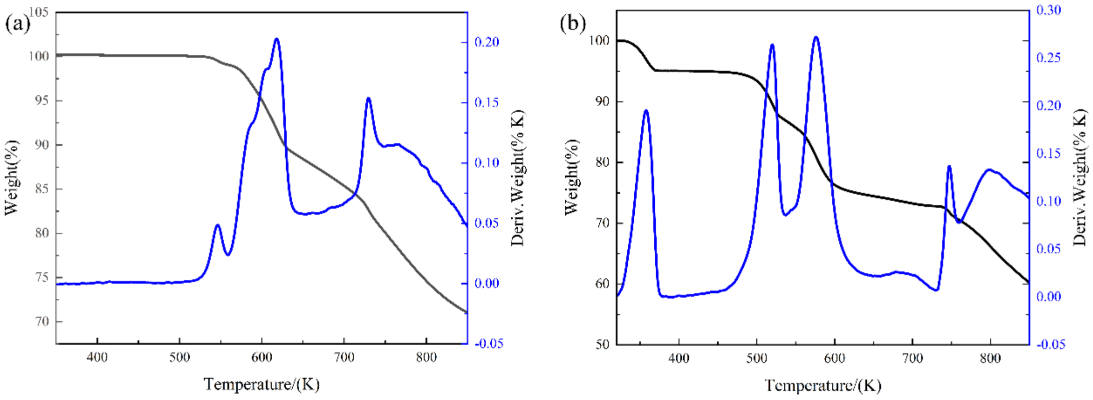

Figure S7 TG test diagram of (a) compound **1** and (b) compound **2**

The thermal stability test of compound 1 was carried out in the temperature range of 350 K-850 K. The DTA and TG test curves of compound **1** were obtained at a heating rate of 10 K / min under nitrogen protection as shown in figure S7 (a). The DTA curve in the figure shows that the weight loss of the compound is divided into three stages, indicating that compound **1** has good thermal stability. The TG curve showed that the compound began to decompose at 527 K. In the first stage, an imidazole molecule was lost in the process of 527 K-628 K, and the weight loss was about 9.8 %, which was basically consistent with the theoretical value of 9.3 %. In the second

stage, the mass loss of an imidazole molecule between 628 K and 724 K is about 8.5 %, which is basically consistent with the theoretical value of 9.3 %. After the third stage is 724 K, the isopolyanion skeleton of compound **1** gradually collapses after this stage, and no longer decomposes after forming a stable oxide structure. It is proved that the thermal decomposition process of the compound is consistent with the crystal structure, infrared spectrum and XRD spectrum.

The DTA and TG curves of compound **2** measured under the same conditions are shown in figure S7 (b). It can be preliminarily judged from the DTA curve that the decomposition of compound **2** is divided into four stages. The TG curve of compound **2** shows that the decomposition of the compound is mainly divided into three stages, and the compound begins to decompose at 331 K. The first stage is between 331 K-370 K, and the actual weight loss value is about 4.3 %. In this stage, two water molecules are mainly lost, which is basically consistent with the theoretical value of 3.5 %. The second stage is between 370 K-629 K. In this stage, the actual weight loss is about 25.4 %, which is a continuous decomposition process. In this process, four imidazole molecules are mainly lost, which is basically consistent with the theoretical value of 26.3 %. The third part is the temperature range after 629 K. At this stage, the skeleton of the same polyanion component in compound **2** began to collapse, and the remaining metal elements continued to exist in the form of oxides. It is proved that the thermal decomposition process of the compound is consistent with the crystal structure and XRD spectrum.

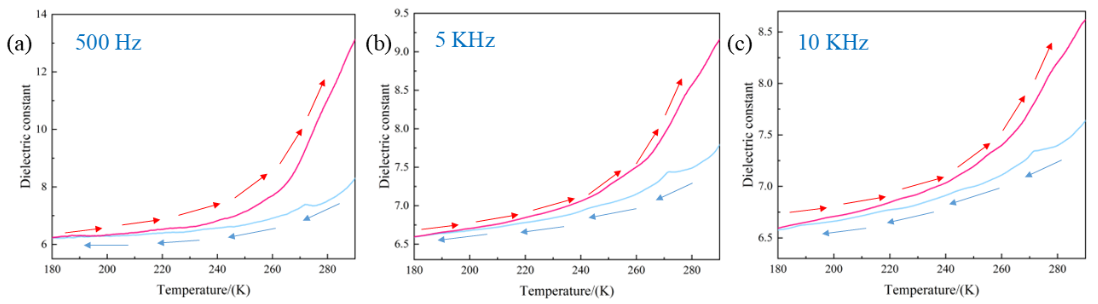

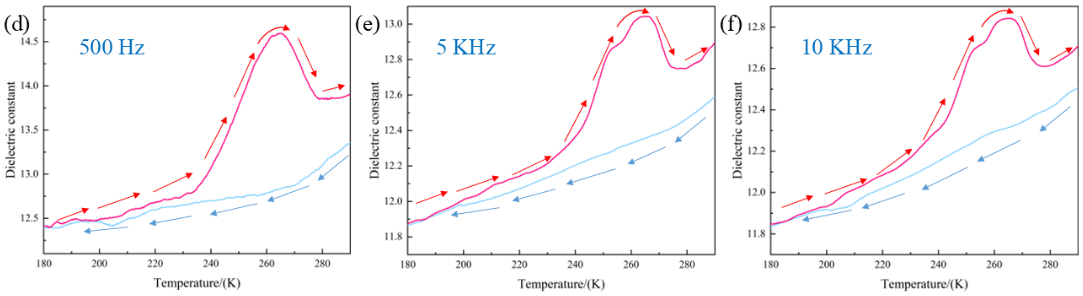

Figure S8 Compounds **1** 500 Hz (a), 5 KHz (b), 10 KHz (c) cyclic dielectric ; compounds **2** 500 Hz (d), 5 KHz (e), 10 KHz (f) cyclic dielectric

The cyclic dielectric test results of compounds **1** and **2** at different frequencies between 180 K-290 K are shown in Figure S8. It is found that these two compounds have good cyclicity at frequencies of 500 Hz, 5 KHz and 10 KHz, respectively, indicating that these two compounds are good dielectric abnormal crystal materials.

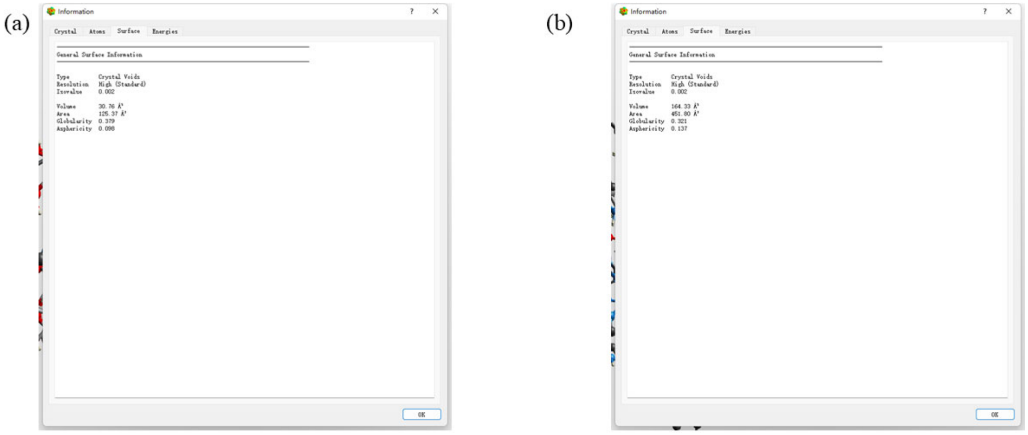

Figure S9 The hole calculation diagram of (a) compound 1 and (b) compound 2

The hollow surfaces of these two compounds were calculated by the Surface Generation module of CrystalExplorer software, and the "hollow" region in the crystal structure could be visualized, as shown in Figure 9. In the software simulation, the void surface of the compound was defined as the isosurface of the electron density of the primary crystal. The whole cell is calculated. And when the surface of the cavity in the cell meets the boundary of the single cell, a cover is generated to create a closed volume. The specific data of the spatial pores of the three compounds were obtained as shown in Figure S7. The pore volumes of the two compounds were 30.76 A^3 and 164.33 A^3, respectively.

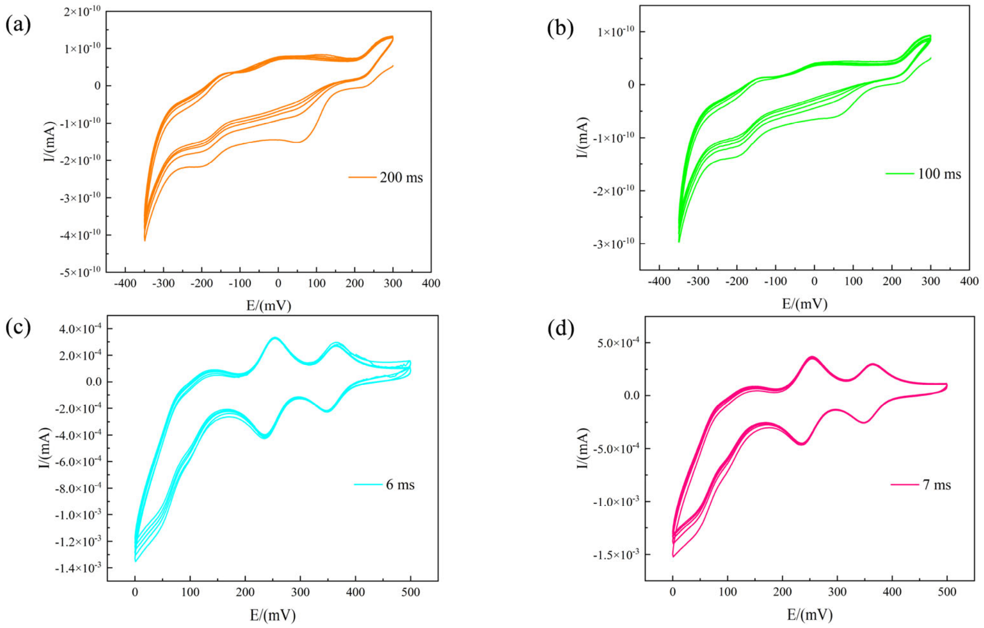

Figure S10 The cyclic test of compound 1 at 100 (a) and 200 ms (b), and the cyclic test of compound 2 at 6 (c) and 7 ms (d).

The cyclic tests of compound 1 and compound 2 at 100, 200 ms and 6 and 7 ms show that the two compounds still maintain good cyclicity after 5 cycles, indicating that the two compounds have good electrochemical stability.
